# Acute Fasting Does Not Induce Cognitive Impairment in Mice

**DOI:** 10.3389/fnins.2019.00896

**Published:** 2019-08-26

**Authors:** Hua Zheng, Hoai Ton, Lei Yang, Ning Liufu, Yuanlin Dong, Yiying Zhang, Zhongcong Xie

**Affiliations:** ^1^Department of Anesthesiology, Tongji Hospital, Tongji Medical College, Huazhong University of Science and Technology, Wuhan, China; ^2^Geriatric Anesthesia Research Unit, Department of Anesthesia, Critical Care and Pain Medicine, Massachusetts General Hospital and Harvard Medical School, Charlestown, MA, United States; ^3^Department of Anesthesiology, Ruijin Hospital, Shanghai Jiaotong University School of Medicine, Shanghai, China; ^4^Department of Anesthesiology, Sun Yat-sen Memorial Hospital, Sun Yat-sen University, Guangzhou, China

**Keywords:** acute fasting, cognitive impairment, neural activation, cellular apoptosis, mice

## Abstract

Preoperative baseline cognitive impairment is associated with postoperative neurocognitive disorder (PND). Fasting, and more generally, calorie restriction has been shown to exert controversial effects in clinical settings and various animal models of neurological disorders. Every patient needs acute fasting before anesthesia and surgery. However, the impact of acute fasting on cognitive function remain largely unknown. We, therefore, set out to determine whether acute fasting can induce neurotoxicity and neurobehavioral deficits in rodents. In the present system establishment study, a mouse model of acute fasting was established. The effects of the acute fasting on natural and learned behavior were evaluated in the buried food test, open field test and the Y maze test. The expression of c-Fos, the marker of neuronal activation, and caspase-3 activation, the marker of cellular apoptosis, were measured with immunohistochemistry. We found that the 9 h acute fasting increased the latency to eat food in the buried food test. The acute fasting also selectively increased the total distance and decreased the freezing time in open field test, and increased the duration in the novel arm in the Y maze test. Besides, the immunohistochemical study showed that the fasting significantly increased the c-Fos level in the hippocampus and various sub-cortical areas, including paraventricular thalamus (PVT), dorsomedial hypothalamus (DMH), lateral hypothalamus (LH), and basal amygdala (BMA). However, the acute fasting did not induce apoptosis, demonstrating by no appearance of caspase-3 activation in the corresponding brain areas. These data showed that acute fasting did not cause cellular apoptosis and cognitive impairment in the mice. Instead, the acute fasting increased the neuronal activity, enhanced the ambulatory activity and improved the spatial recognition memory in the mice. These findings will promote more research in the established system to further determine the effects of perioperative factors on the postoperative neurocognitive function and the underlying mechanisms.

## Introduction

Postoperative neurocognitive disorder (PND), including postoperative cognitive dysfunction and postoperative delirium, is one of the most common postoperative complications in elderly patients and is associated with increased morbidity, mortality and costs of medical care (Gleason et al., 2015; Crocker et al., 2016; Inouye et al., 2016; Marcantonio, 2017). However, its causes, neuropathogenesis, and risk factors remain largely unknown. Several risk factors are identified to contribute to the PND, including preoperative baseline cognitive impairment (Robinson et al., 2012; Vasilevskis et al., 2012; Silbert et al., 2015; Raats et al., 2016). Specifically, a narrative review of 54 studies has shown that preoperative baseline cognitive impairment is associated with 2- to 17-fold increases in PND (Oresanya et al., 2014). Therefore, it is essential to reduce any risk factors associated with the preoperative baseline cognitive impairment.

Clinically, the patients usually have more than 12 h fasting before their anesthesia and surgery. Fasting, and more generally, calorie restriction has been shown to exert controversial outcomes (Benau et al., 2014; Longchamp et al., 2017). In various animal models of neurological diseases, such as epilepsy, Parkinson’s disease and Alzheimer’s disease (Greene et al., 2001; Maswood et al., 2004; Halagappa et al., 2007), calorie restriction is reported to mitigate the neurological damage. In healthy human intervention trials, intermittent fasting appears to results in weight loss with mixed impacts on metabolic biomarkers associated with risk of cardiovascular disease, diabetes, and cancer (Patterson et al., 2015). In clinical settings, preoperative fasting initiates the metabolic stress response, leads to hyperglycemia and insulin resistance, which is associated with postoperative complications (e.g., impaired wound healing and immunosuppression) and the length of hospital stay (Melnyk et al., 2011). However, the effects of acute fasting on cognitive function remain largely to be determined. We, therefore, set out to assess the impact of acute fasting on the cognitive function in mice. The outcomes of the present studies would ultimately illustrate whether acute fasting is a risk factor of the PND.

C-Fos is one of the immediate early genes, which are the first group of genes expressed after stimulation of neurons (Gallo et al., 2018). The basal expression of c-Fos is at a relatively low level in brains. Elevated levels of c-Fos transcription can be induced by calcium influxes resulting from extracellular stimulation (Morgan and Curran, 1986). Thus, the imaging of c-Fos is considered to be a marker of stimulation-related neural activation and is often used as a method of visualizing neuronal networks that are activated (Kemp et al., 2013). In the present studies, we assessed the effects of acute fasting on the brain levels of c-Fos, the marker of neuronal activation, and caspase-3 activation, the marker of cellular apoptosis (Crowley and Waterhouse, 2016; Lossi et al., 2018).

The objective of the current study was to determine the effects of acute fasting on the cognitive function in adult mice and the cellular changes including caspase-3 activation and c-Fos expression in the brain tissues of the mice. The hypothesis in the present study was that acute fasting induced cognitive impairment, brain c-Fos expression, and caspase-3 activation in mice. Specifically, we used a battery of behavioral tests (buried food test, open field test, and Y maze test) (Peng et al., 2016) to determine the behavioral changes in 9 month-old mice. We further assessed the effects of acute fasting on the c-Fos levels in different brain regions (e.g., cortex and hippocampus) and on the levels of caspase-3 in the brain tissues of the mice.

## Materials and Methods

### Animals and Acute Fasting Model

The protocol of the animal study was approved by the Massachusetts General Hospital Standing Committee on Animals (Boston, MA, United States) on the use of Animals in Research and Teaching. We performed animal studies according to the regulation of the National Institutes of Health (Bethesda, MD, United States). All efforts were used to reduce the number of mice used in the experiments. C57BL/6J female mice (wild-type) were used in the experiments. The C57BL/6 mice (9-month-old) were obtained from Jackson Labs (Bar Harbor, ME, United States). The mice were housed in a temperate- and humidity-controlled environment (20–22°C; 12-h light: dark on a reversed light cycle; lights-off at 7:00 p.m.) with free access to water and food (control mice), and to water but not food (acute fasting mice). Mice were randomly assigned to one of two groups: control (fed *ad libitum*) and fasting (food removed). In the fasting group, the mice were fasted by completely depriving of food for 9 h, which was started at 8:00 a.m. In the control group, the mice had free access to food. Water was available *ad libitum* for the mice in the two groups. In the current system establishment study, we only used female mice.

### Behavioral Tests

**FIGURE 1:**
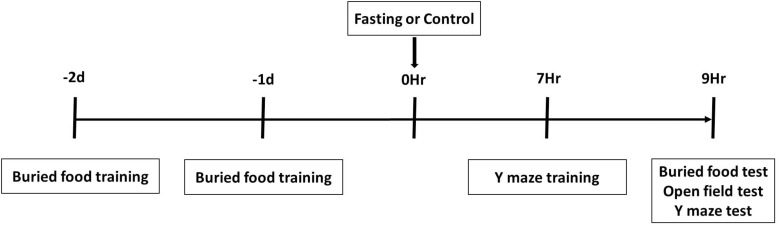
Schematic diagram of experimental design. The mice receive buried food training at 2 days and 1 day before treatment (fasting or control) and Y maze training at 7 h after treatment, and then behavior tests in the order of buried food test, open field test and Y maze test at 9 h after treatment.

A battery of behavioral tests including buried food test, followed by an open field test and Y maze test, were performed as described in our previous study (Peng et al., 2016) with modifications. As demonstrated in the diagram (Figure 1), all mice had buried food training at 2 days and 1 day before fasting or control condition, and had Y maze training at 7 h after the fasting or control condition. The mice then had the behavioral tests in the order of buried food test, open field test and Y maze test at 9 h after fasting or control condition. We performed multiple behavior tests in groups of 4 mice and finished the tests in these mice within 1 h.

#### Buried Food Test

Before the test, all mice were familiarized with sweetened cereal (Kellogg’s Froot Loops, Battle Creek, MI, United States) daily over the 2 days before the testing. During the test, a mouse was placed in the center of a testing cage (30 cm × 19 cm × 13 cm, width × length × height) to seek and recover one piece of sweetened cereal, which was buried 0.5 cm below the surface of a 3 cm deep layer of fresh bedding. The time between the placement of the mouse in the testing cage and the time when the mouse grasped the sweetened cereal with its forepaws and teeth was recorded and defined as the latency time of buried food test. If a mouse failed to locate the food within 300 s, the testing session ended and the latency time was recorded as 300 s. Mice were allowed to consume the sweetened cereal and then returned to their home cage. After the test, the testing cage was cleaned with 70% ethanol solution, and the bedding inside was changed between the trials.

#### Open Field Test

The open field test was performed in a square arena (40 cm × 40 cm × 40 cm, width × length × height), with an open top, four white walls, and a gray floor. After a mouse was placed in the corner of the open field, the ambulation of the mouse for 5 min was recorded by a video tracking software (Any-Maze behavior tracking software, Stoelting Co., Wood Dale, IL, United States). The total distance traveled (m), the freezing time (seconds), the latency to the center (seconds), and the time spent in the center of the open field (seconds) were analyzed. After the test, mice were removed from the open field and returned to their home cage. The floor of the apparatus was cleaned with 70% ethanol solution before the next testing.

#### Y Maze Test

The Y maze test was performed in a Y-shaped maze, which consisted of three gray polyvinylene arms (8 cm × 30 cm × 15 cm, width × length × height), with a 120° angle from each other. Three arms were randomly designated as start arm, novel arm, and other arm. The Y-maze test included two trials separated by a 2-h interval. In the first trial (training), the novel arm was blocked, and the mouse was allowed to explore the start arm and other arm for 10 min. In the second trial (retention), all three arms were opened, and the mouse was allowed to move freely for 5 min from the end of the same start arm. A ceiling-mounted video camera and Any-Maze behavior tracking software were used to record and analyze the number of arm entries and the time spent in each arm. Recognition of the novel arm from the start arm and other arm is considered as the spatial recognition memory (learned behavior). Maze arms were cleaned with 70% ethanol solution between trials.

### Immunohistochemistry

A different group of mice was used for immunohistochemistry studies. After 9 h of fasting or *ad libitum*, the mice were anesthetized with 1.4% isoflurane for 3 min and perfused transcardially with PBS followed by 4% cold, buffered paraformaldehyde. The brain tissues were removed and post-fixed at 4°C overnight, and then cryoprotected in 15% sucrose followed by 30% sucrose in PBS for 48 h. 20-μm coronal sections were obtained with a Leica CM1850 cryostat. Non-specific antibody binding was inhibited by incubating the slices in 0.1 M PBS containing 10% goat serum and 0.3% Triton X-100. Slices were then incubated for 24 h at 4°C with one of the following primary antibodies: rabbit anti-c-Fos antibody (#2250, 1:200 dilution, Cell Signaling Technology, Danvers, MA, United States), rabbit anti-caspase-3 antibody (#9662, 1:200 dilution, Cell Signaling Technology, Danvers, MA, United States), and rabbit anti-cleaved caspase-3 antibody (#9661, 1:200 dilution, Cell Signaling Technology, Danvers, MA, United States). After the incubation, tissue sections were washed and incubated for 1 h at room temperature with Alexa Fluor 488 or Alexa Fluor 594 (1:500 dilution, Thermo Fisher Scientific, Rockford, IL, United States) as the secondary antibodies. Fluoroshield medium with DAPI (ab104139, Abcam, Cambridge, MA, United States) was used for nuclear counterstain.

### Quantification

Tissue sections were examined under an All-in-One Keyence BZ-9000 microscope interfaced to a computer workstation. We used the Mouse Brain Atlas (Paxinos and Franklin, 2001) to visually guide localization of the regions of interest (ROI). The brain regions at bregma −1.7 ± 0.2 mm that were examined in all of the groups are diagrammed in Figures 5D, 6B. Digital images of coronal sections containing the following brain areas were captured by a BZ-II Viewer program: dentate gyrus (DG), CA1, CA2/3, paraventricular thalamus (PVT), dorsomedial hypothalamus (DMH), lateral hypothalamus (LH), and basal amygdala (BMA). All cells with staining intensities that were ≥2 × background were quantified manually at 200× magnification using the Cell Counter function in BZ-II Analyzer.

### Statistics

The number of samples was 12–16 per group in the behavioral tests, and 4–6 per group in the immunohistochemical studies. Data of behavior tests and immunohistochemistry were presented as the mean ± standard error of the mean (SEM). Normality of data was first analyzed by using the Shapiro-Wilk test. The latency to eat food in the buried food test, the latency to the center, time spent in the center and freezing time in the open field test were found not normally distributed. Thus, the data were analyzed by the Mann-Whitney test. Normally distributed data, including the total distance moved in the open field test, the number of arm entries, entries, and duration in novel arm in Y maze test and immunohistochemical results, were analyzed by the Student’s *t*-test. Statistical comparisons were deemed significantly different if *p* < 0.05. Graphpad Prism 6.0 (GraphPad Software, Inc., La Jolla, CA, United States) was used for all analyses.

## Results

### Acute Fasting Enhanced the Natural Behavior of Mice in the Buried Food Test and Open Field Test

**FIGURE 2:**
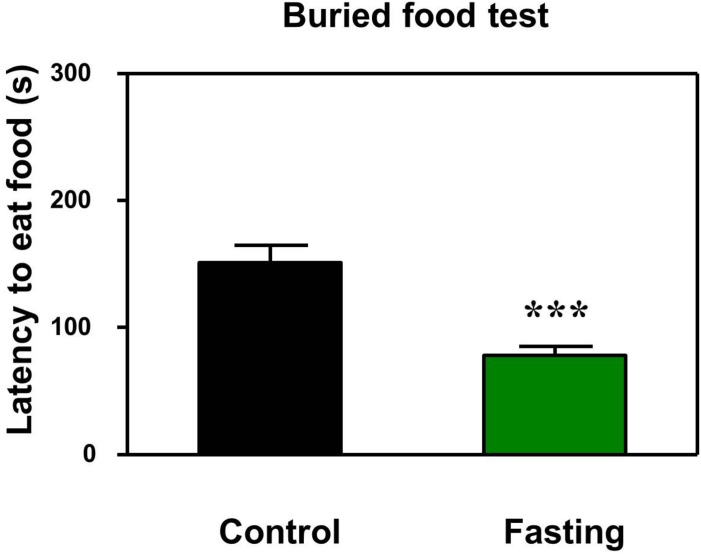
Acute fasting enhances the natural behavior of mice in buried food test. The bar graphs show that acute fasting for 9 h (green column) significantly decreases the latency to eat the food of the mice as compared to the control condition (black column) in the buried food test. Data are shown as means ± SEM (Mann-Whitney test; ^∗∗∗^*p* < 0.001; *n* = 12–16 animals).

**FIGURE 3:**
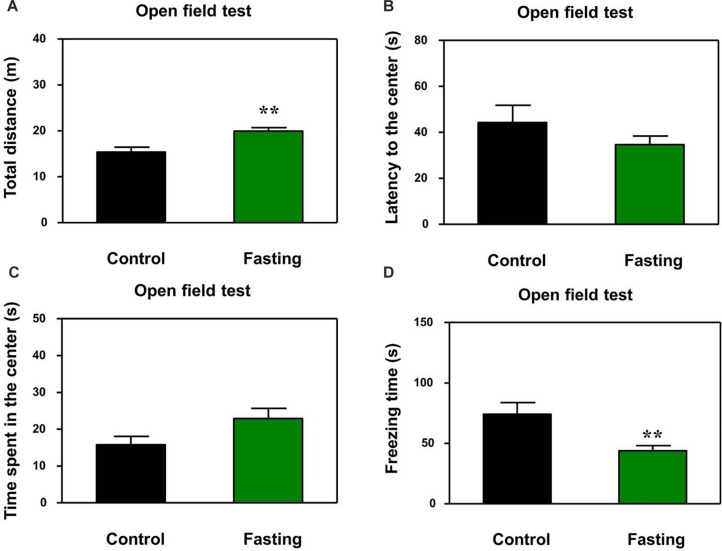
Acute fasting enhances the natural behavior of mice in the open field test. The bar graphs show the total distance moved **(A)**, the latency to the center **(B)**, time spent in the center of the open field **(C)**, and freezing time **(D)** of mice in control (black columns) and fasting groups (green columns). Note that 9-h fasting significantly elevated the distance traveled and reduced the freezing time as compared to the control condition. Data are shown as means ± SEM (Mann-Whitney test or Student’s *t*-test; ^∗∗^*p* < 0.01; *n* = 12–16 animals).

First, we performed the buried food test and open field test in the mice to assess the changes in natural behavior of the mice after the 9 h fasting. As can be seen in Figure 2, the acute fasting for 9 h decreased the latency of mice to eat the food as compared to the control condition (*p* < 0.001, Figure 2). Then as shown in Figure 3, there was no significant difference in the latency to the center (*p* = 0.416; Figure 3B) and the central zone activities (*p* = 0.061; Figure 3C) between the mice in the fasting group and the mice in the control group in the open field test. However, the acute fasting significantly increased the distance traveled (19.95 ± 0.77 vs. 15.42 ± 1.07, *p* = 0.001; Figure 3A) while decreased the freezing time (44.00 ± 4.07 vs. 74.18 ± 9.68, *p* = 0.006; Figure 3D) as compared to the control condition in the mice. Collectively, these data demonstrated that the acute fasting would enhance the natural behavior of the mice.

### Acute Fasting Enhanced the Learned Behavior of Mice in the Y-Maze Test

**FIGURE 4:**
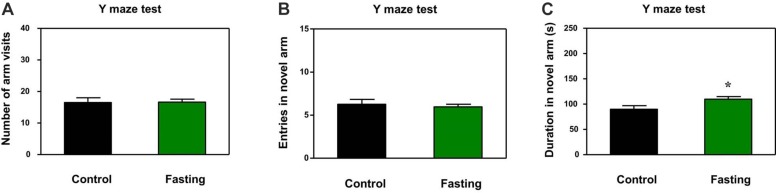
Acute fasting enhances the learned behavior of mice in the Y-maze test. The graphs are showing the number of arm entries **(A)**, novel arm entries **(B)** and duration in the novel arm **(C)**. Note that 9-h fasting significantly elevated the duration in the novel arm as compared to the control condition. Data are shown as means ± SEM (Student’s *t*-test; ^∗^*p* < 0.05; *n* = 12–16 animals).

In addition to natural behavior, learned behavior of mice was assessed by using the Y-maze test after the 9 h fasting. There was no significant difference in the number of arm entries (*p* = 0.945; Figure 4A) and novel arm entries (*p* = 0.641; Figure 4B) between the mice in the fasting group and the mice in the control group. However, the acute fasting increased the time spent in open arms of the mice as compared to the control condition (109.90 ± 5.19 vs. 89.91 ± 7.04, *p* = 0.024; Figure 4C). In the current study, the numbers of arm entries were not significantly different between the mice in the fasting group and the mice in the control group, suggesting that the more time spent in the open arms by the mice in the fasting group as compared to the mice in the control group may not be due to the changes in the locomotor activity of the mice. These data demonstrated that the acute fasting enhanced the spatial recognition memory in the mice.

### Acute Fasting Increased the C-Fos Levels in Brain Tissues of the Mice

**FIGURE 5:**
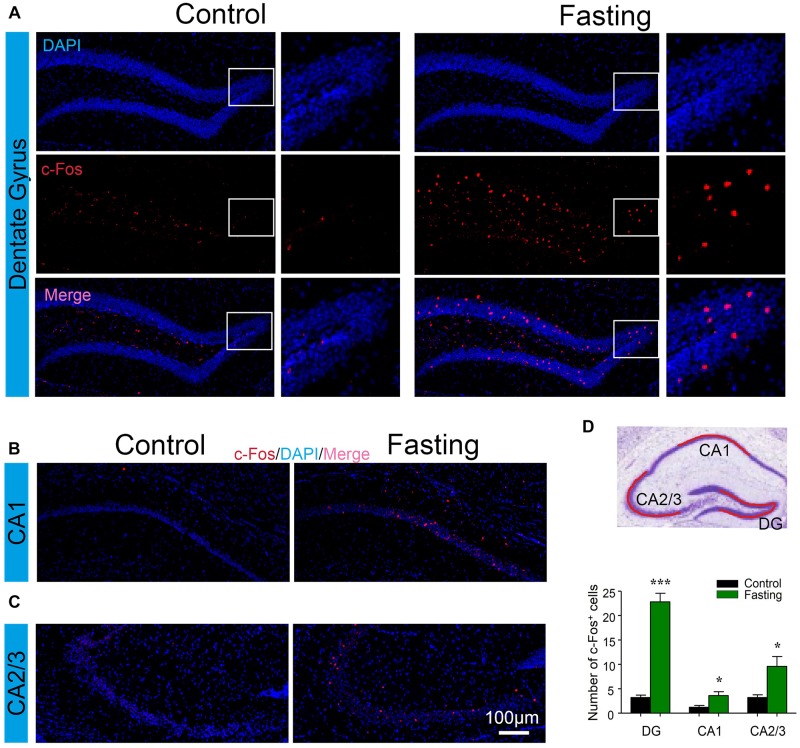
Acute fasting increases the c-Fos expression in different sub-hippocampal areas. **(A–C)** Representative photomicrographs of c-Fos immunostaining in the dentate gyrus (DG), CA1, and CA2/3. There were a higher number of c-Fos^+^ cells in the fasting group (right panel) in comparison with the control group (left panel). Scale bars represent 100 μm. **(D)** Schematic illustration of the hippocampus. The red line indicates the captured and analyzed sub-region of hippocampus including DG, CA1, and CA2/3 areas. The bar graph shows the number of c-Fos^+^ cells in different hippocampal areas. Note that the number of c-Fos^+^ cells was significantly increased following 9-h fasting as compared to the control condition. Data are shown as mean ± SEM (Student’s *t*-test; ^∗^*p* < 0.05; ^∗∗∗^*p* < 0.001; *n* = 4–6 animals).

Next, to explore which specific brain regions were associated in the behavioral changes induced by acute fasting, we investigated c-Fos expression in different brain areas. The hippocampus has been studied extensively as part of a brain system responsible for learning and memory (Jeffery, 2018). To determine whether the acute fasting can affect the c-Fos level in the hippocampus, we performed c-Fos immunostaining and counted c-Fos^+^ cells in the DG, CA1, and CA2/3 areas. As can be seen in Figure 5, there were increased numbers of c-Fos^+^ cells in the DG of the mice in the fasting group as compared to the mice in the control group (22.80 ± 1.77 vs. 3.20 ± 0.49, *p* < 0.001; Figure 5A). Similarly, there were more c-Fos^+^ cells in the CA1 (3.60 ± 0.81 vs. 1.20 ± 0.37, *p* = 0.028; Figure 5B) and CA2/3 (9.60 ± 2.02 vs. 3.20 ± 0.58, *p* = 0.016; Figure 5C) of the mice in the fasting group as compared to the mice in the control group. These data showed that acute fasting significantly increased the number of c-Fos^+^ cells in the hippocampus of the mice.

**FIGURE 6:**
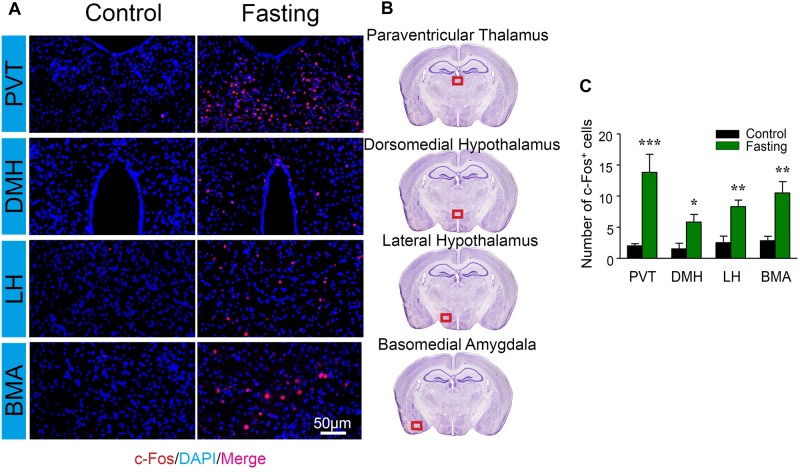
Acute fasting increases the c-Fos expression in different sub-cortical areas. **(A)** Representative photomicrographs of c-Fos immunostaining in the sub-region of the cortex including paraventricular thalamus (PVT), dorsomedial hypothalamus (DMH), lateral hypothalamus (LH), and basal amygdala (BMA). There were a higher number of c-Fos^+^ cells in the fasting group (right panel) in comparison with the control group (left panel). Scale bars represent 50 μm. **(B)** Schematic illustration of the regions of interest. The red box (500 μm × 140 μm) indicates the captured and analyzed sub-region of the cortex. **(C)** The number of c-Fos^+^ cells in different sub-cortical areas. Note that the number of c-Fos^+^ cells was significantly elevated following 9-h fasting as compared to the control condition. Data are shown as mean ± SEM (Student’s *t*-test; ^∗^*p* < 0.05; ^∗∗^*p* < 0.01; ^∗∗∗^*p* < 0.001; *n* = 4–6 animals).

We further characterized c-Fos expression in other cognitive-related brain regions. There were increased number of c-Fos^+^ cells in PVT (13.83 ± 2.89 vs. 2.00 ± 0.35, *p* < 0.001; Figure 6), DMH (5.83 ± 1.22 vs. 1.50 ± 0.92, *p* = 0.018; Figure 6), LH (8.33 ± 1.02 vs. 2.50 ± 1.06, *p* = 0.003; Figure 6), and the BMA (10.50 ± 1.82 vs. 2.83 ± 0.70, *p* = 0.003; Figure 6) of the mice in the fasting group as compared to the mice in the control group. Collectively, these data demonstrated that acute fasting was associated with significantly increased levels of c-Fos protein in specific sub-regions of thalamus, hypothalamus, and amygdala.

### Acute Fasting Did Not Induce Cell Apoptosis in Sub-Hippocampal and Sub-Cortical Areas

**FIGURE 7:**
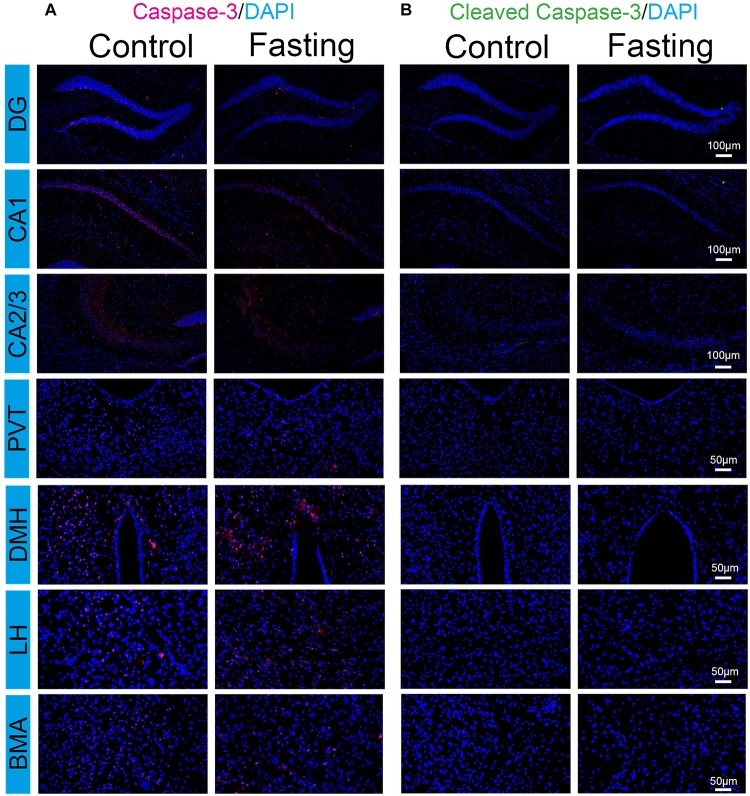
Acute fasting does not induce cell apoptosis in sub-hippocampal and sub-cortical areas. Representative photomicrographs of caspace-3 **(A)** and cleaved caspace-3 **(B)** immunostaining in the sub-region of the hippocampus and cortex including DG, CA1 and CA2/3, PVT, DMH, LH, and BMA. No cleaved caspase-3 immunoreactivity was detected in both control and fasting group. Scale bars, 100 μm (DG, CA1, and CA2/3), 50 μm (PVT, DMH, LH, and BMA).

The increased c-Fos expression has been shown to associate with increased neuronal activity. However, c-Fos overexpression could also play a causal role in cell death (Preston et al., 1996; Inada et al., 1998). Cleaved caspase-3 immunoreactivity is a marker of cells undergoing apoptosis. We, therefore, used frozen coronal brain sections to detect the cleaved (activated) caspase-3 by using immunohistochemical analysis. The cell counts from each mouse were obtained from the same areas corresponding to the region of interest (ROI) of the counted c-Fos from a standard mouse brain atlas (Paxinos and Franklin, 2001). As shown in Figure 7, total caspase-3 immunoreactivity in the brain regions were observed in the mice of both control and fasting groups without significant difference. In contrast, cleaved caspase-3 immunoreactivity was not detected despite prominent c-Fos immunoreactivity in these regions. Moreover, we found that sleep disturbance increased the number of the cleaved caspase-3 positive cells in several brain regions in the mice (Supplementary Figure S1), consistent with the findings of our previous studies (Zhu et al., 2012) and suggesting that the cleaved caspase-3 antibody in the current study was able to demonstrate the activation of caspase-3 in the brain tissues of mice. Taken together, these data showed that the observed robust increases in c-Fos immunoreactivity induced by fasting were not associated with the cellular apoptosis in the brain of the mice following acute fasting in the mice.

## Discussion

In the current studies, we showed that 9 h of acute fasting did not impair both natural behavior and learned behavior in mice. Besides, the acute fasting increased the c-Fos protein expression in several sub-regions in the brain of the mice. Finally, the acute fasting did not induce apoptosis, demonstrating by no activation of caspase-3 in the corresponding brain areas. These results did not support our hypothesis. Instead, these data showed that the 9 h acute fasting did not induce neurotoxicity and neurobehavioral deficits in the mice.

We first found that the 9 h acute fasting decreased the latency of mice to eat the food as compared to the control condition in the buried food test (Figure 2). The buried food test assess the olfactory function and odor discrimination of rodent. However, the mouse should have intact cognitive ability (e.g., attention) to complete the buried food test. Thus, the buried food test in the present study also assessed the cognitive function together with the open field test and Y maze test. Next, our results showed that the 9 h acute fasting increased the total distance traveled as compared to the control condition in the mice in the open field test (Figure 3A). The total distance traveled by mice represents the locomotor activity of the mice (Sestakova et al., 2013), indicating that the acute fasting in the current study increased the locomotor activity of the mice. Then, the finding that the acute fasting decreased the freezing time in the open field test as compared to the control condition (Figure 3D) showed that the acute fasting altered the anxiety-like behaviors of the mice (Prut and Belzung, 2003; Chen et al., 2014). Finally, we observed that acute fasting increased the duration in the novel arm in the mice (Figure 4C). These results indicated that the acute fasting enhanced the spatial recognition memory of the mice as compared to the control condition, consistent with the findings from the other studies (Rayatnia et al., 2011; Wolf et al., 2016). Taken together, the 9 h acute fasting was able to improve certain natural and learned behaviors associated with cognitive function in mice.

In the current study, we observed the increased activity in the mice after 9 h of acute fasting. Consistently, in the studies by Overton and Williams (2004) and Kanizsai et al. (2009), the mice also exhibited increased locomotor activity after fasting or caloric restriction. Besides, the increased locomotor activity in the mice disappeared when the ambient temperature was increased to 29–33°C (Overton and Williams, 2004). These findings were consistent with the observation that the increased locomotor activity of the mice occurred in the temperature of 20–22°C, potentially resulting in some cold-associated stress. Future studies to investigate the interaction of acute fasting and body temperature on the potential neurotoxicity and neurobehavioral deficits are warranted. Interestingly, behavioral activity during fasting has also been shown either to remain unchanged or to be decreased in mice (Gelegen et al., 2006; Tucci et al., 2006). However, these studies were different from our current studies. First, there was a variety of fasting durations. The duration of fasting was reported to be an essential variable in determining the effects of acute fasting on the behavior of mice (Cui et al., 2018). The duration of fasting in other studies ranged from days to weeks, but the duration of the fasting in the current studies was 9 h. Second, the age of mice used was different. There is evidence to suggest that aging affects behaviors such as exploration activity and object recognition in mice (Fahlstrom et al., 2012). The mice in other studies were 2–6 months old. However, the age of mice in the current study was 9 month old. Third, the behavioral tests utilized in the studies were diverse. In other studies, the locomotor activity was detected through a single test. In the current studies, a battery of behavioral test was performed to assess changes in both natural and learned behavior in the mice.

We selected 9 h, not longer time, of acute fasting to minimize animal suffering (Cui et al., 2018). The outcomes from the current studies suggest that the 9 h acute fasting did not induce cognitive impairment in the 9 month-old wild-type mice. However, it is still possible that the acute fasting might have different effects in the more vulnerable mice, such as aged mice and Alzheimer’s disease transgenic mice. Future studies using the established system to determine the impact of acute fasting on cognitive function and the associated cellular changes in both aged and Alzheimer’s disease transgenic mice are warranted.

The increases in c-Fos expression have been shown in several stimuli models including fasting (Gallo et al., 2018). In the mechanistic studies, the increased c-Fos expression was observed in many brain areas following the acute fasting. Specifically, the increased c-Fos expression by acute fasting has been reported in the prefrontal cortex (Lutter et al., 2008), amygdala (Velazquez et al., 2015), and hypothalamus (Cano et al., 2003; Riediger et al., 2004). However, the current study was the first one to systemically investigate the c-Fos expression in many sub-regions of brains involved in the behavioral changes in motivated animals induced by fasting. C-Fos expression induced by the 9 h acute fasting was significantly enhanced in many brain areas. These data indicated the substantial impacts of acute fasting on the neural system. Interestingly, most remarkable c-Fos activation was observed in the DG of the hippocampus (Figure 5A) and PVT (Figure 6A). These data revealed that DG and PVT might be the essential brain regions on the mechanism of the behavioral changes induced by acute fasting. The fasting-related neural circuit and its associated behavioral changes are worthy of further investigation in the future.

Moreover, we evaluated the level of caspase-3 and cleaved caspase-3, the biomarkers of cellular apoptosis (Crowley and Waterhouse, 2016; Lossi et al., 2018), in the mice in the acute fasting group or the mice in the control group. We found that cleaved caspase-3 immunoreactivity was detected in neither the mice in the control group nor the mice in the fasting group (Figure 7B). However, the c-Fos immunoreactivity was prominent in the brain regions of the mice in the acute fasting group. These data demonstrated that acute fasting increased neuronal activities but did not induce apoptosis in the particular brain areas associated with cognitive function.

There are several limitations in the current studies. First, each of the mice in the fasting group received the three behavioral tests in the sequence of buried food test, open field test and Y maze test. Therefore, the earlier behavioral test could interfere the following behavioral test. In order to minimize such interference, we purposely performed the natural behavioral tests (buried food test and open field test) before the learned behavioral test (Y-maze test). Moreover, the mouse in the control group received the same behavioral tests with the same sequence. Second, we only used female mice (C57BL/6J, wild-type, 9-month-old) in the present study to establish the system and demonstrate the effects of fasting on the behavioral and biochemistry changes in the mice. We will use the established system to determine the sex-dependent changes in the future. Third, an increased expression of c-Fos after acute fasting has been shown in this study. However, the mechanism of this induction and whether the induction of c-Fos is neuroprotective or detrimental to the brain after fasting is not clear. Nevertheless, the outcomes showed that acute fasting might influence neuronal physiology but might not cause neurotoxicity. Forth, the cause-effect link between the fasting-induced changes in c-Fos expression and the fasting-induced behavioral changes also remain uncovered. However, the current studies have established a system to perform such research in the future.

Postoperative neurocognitive disorder is a common postoperative complication (Vutskits and Xie, 2016). However, the causes and neuropathogenesis of PND remain largely to be determined. In clinical settings, every patient needs to have fasting before anesthesia, and surgery to avoid potential aspiration of food and water into lungs. Therefore, it is important to know whether fasting can induce neurotoxicity and neurobehavioral deficits. The finding in the current studies showed that acute fasting induced neither neuronal apoptosis nor cognitive impairment in adult mice. These results suggest that fasting may not be the risk factor of PND, pending further investigations. However, it is still not known whether fasting in young and aged mice, which are more vulnerable to the development to PND, can cause neurotoxicity and neurobehavioral deficits. We will use the established system to answer these questions in the future studies.

## Conclusion

In conclusion, we found that the 9 h acute fasting improved the natural and learned behavior in mice. Besides, acute fasting increased the number of c-Fos^+^ cells in various brain areas without causing caspase-3 activation. These results suggest that acute fasting in mice might not cause neurotoxicity and cognitive impairment in mice. These findings would promote further investigation to determine the effects of perioperative factors, e.g., acute fasting, on the PND in the future.

## Data Availability

The datasets generated for this study are available on request to the corresponding author.

## Author Contributions

HZ, HT, and ZX designed the experiments. HZ and NL performed the behavioral tests. HZ and LY conducted the immunohistochemical studies. HZ, HT, YD, and YZ analyzed the data. HZ, HT, and ZX wrote the manuscript.

## Conflict of Interest Statement

The authors declare that the research was conducted in the absence of any commercial or financial relationships that could be construed as a potential conflict of interest.

## Supplementary Material

FIGURE S1 Positive control of immunohistochemistry stained with anti-cleaved caspase-3 antibody. The representative immunohistochemical staining is showing that sleep disturbance -induced cleaved caspase-3 expression distribute in the cortex of mice. The coronal section is located 1.2 ± 0.2 mm anterior from the bregma. The region of interest is outlined with a dash-dot line. Arrows indicate cleaved caspase-3-positive neurons. Scale bars, 100 μm (left image), 50 μm (right image).

## References

[B1] Benau E. M. Orloff N. C. Janke E. A. Serpell L. Timko C. A. (2014). A systematic review of the effects of experimental fasting on cognition. *Appetite* 77 52–61. 10.1016/j.appet.2014.02.014 24583414

[B2] Cano V. Ezquerra L. Ramos M. P. Ruiz-Gayo M. (2003). Characterization of the role of endogenous cholecystokinin on the activity of the paraventricular nucleus of the hypothalamus in rats. *Br. J. Pharmacol.* 140 964–970. 10.1038/sj.bjp.0705513 14517181 PMC1574103

[B3] Chen Y. Liu X. Jia X. Zong W. Ma Y. Xu F. (2014). Anxiety- and depressive-like behaviors in olfactory deficient Cnga2 knockout mice. *Behav. Brain Res.* 275 219–224. 10.1016/j.bbr.2014.08.042 25192635

[B4] Crocker E. Beggs T. Hassan A. Denault A. Lamarche Y. Bagshaw S. (2016). Long-term effects of postoperative delirium in patients undergoing cardiac operation: a systematic review. *Ann. Thorac. Surg.* 102 1391–1399. 10.1016/j.athoracsur.2016.04.071 27344279

[B5] Crowley L. C. Waterhouse N. J. (2016). Detecting cleaved caspase-3 in apoptotic cells by flow cytometry. *Cold Spring Harb Protoc.* 2016. 10.1101/pdb.prot087312 27803251

[B6] Cui R. Fan J. Ge T. Tang L. Li B. (2018). The mechanism of acute fasting-induced antidepressant-like effects in mice. *J. Cell Mol. Med.* 22 223–229. 10.1111/jcmm.13310 28782175 PMC5742683

[B7] Fahlstrom A. Zeberg H. Ulfhake B. (2012). Changes in behaviors of male C57BL/6J mice across adult life span and effects of dietary restriction. *Age* 34 1435–1452. 10.1007/s11357-011-9320-7 21989972 PMC3528371

[B8] Gallo F. T. Katche C. Morici J. F. Medina J. H. Weisstaub N. V. (2018). Immediate early genes, memory and psychiatric disorders: focus on c-Fos, Egr1 and Arc. *Front. Behav. Neurosci.* 12:79. 10.3389/fnbeh.2018.00079 29755331 PMC5932360

[B9] Gelegen C. Collier D. A. Campbell I. C. Oppelaar H. Kas M. J. (2006). Behavioral, physiological, and molecular differences in response to dietary restriction in three inbred mouse strains. *Am. J. Physiol. Endocrinol. Metab.* 291 E574–E581. 10.1152/ajpendo.00068.2006 16670152

[B10] Gleason L. J. Schmitt E. M. Kosar C. M. Tabloski P. Saczynski J. S. Robinson T. (2015). Effect of delirium and other major complications on outcomes after elective surgery in older adults. *JAMA Surg.* 150 1134–1140. 10.1001/jamasurg.2015.2606 26352694 PMC4684425

[B11] Greene A. E. Todorova M. T. McGowan R. Seyfried T. N. (2001). Caloric restriction inhibits seizure susceptibility in epileptic EL mice by reducing blood glucose. *Epilepsia* 42 1371–1378. 10.1046/j.1528-1157.2001.17601.x 11879337

[B12] Halagappa V. K. Guo Z. Pearson M. Matsuoka Y. Cutler R. G. Laferla F. M. (2007). Intermittent fasting and caloric restriction ameliorate age-related behavioral deficits in the triple-transgenic mouse model of Alzheimer’s disease. *Neurobiol. Dis.* 26 212–220. 10.1016/j.nbd.2006.12.019 17306982

[B13] Inada K. Okada S. Phuchareon J. Hatano M. Sugimoto T. Moriya H. (1998). c-Fos induces apoptosis in germinal center B cells. *J. Immunol.* 161 3853–3861. 9780150

[B14] Inouye S. K. Marcantonio E. R. Kosar C. M. Tommet D. Schmitt E. M. Travison T. G. (2016). The short-term and long-term relationship between delirium and cognitive trajectory in older surgical patients. *Alzheimers Dement.* 12 766–775. 10.1016/j.jalz.2016.03.005 27103261 PMC4947419

[B15] Jeffery K. J. (2018). The hippocampus: from memory, to map, to memory map. *Trends Neurosci.* 41 64–66. 10.1016/j.tins.2017.12.004 29405927

[B16] Kanizsai P. Garami A. Solymar M. Szolcsanyi J. Szelenyi Z. (2009). Energetics of fasting heterothermia in TRPV1-KO and wild type mice. *Physiol. Behav.* 96 149–154. 10.1016/j.physbeh.2008.09.023 18938188

[B17] Kemp A. Tischmeyer W. Manahan-Vaughan D. (2013). Learning-facilitated long-term depression requires activation of the immediate early gene, c-fos, and is transcription dependent. *Behav. Brain Res.* 254 83–91. 10.1016/j.bbr.2013.04.036 23644186

[B18] Longchamp A. Harputlugil E. Corpataux J. M. Ozaki C. K. Mitchell J. R. (2017). Is overnight fasting before surgery too much or not enough? how basic aging research can guide preoperative nutritional recommendations to improve surgical outcomes: a mini-review. *Gerontology* 63 228–237. 10.1159/000453109 28052287 PMC5722208

[B19] Lossi L. Castagna C. Merighi A. (2018). Caspase-3 mediated cell death in the normal development of the mammalian cerebellum. *Int. J. Mol. Sci.* 19:E3999. 10.3390/ijms19123999 30545052 PMC6321612

[B20] Lutter M. Krishnan V. Russo S. J. Jung S. McClung C. A. Nestler E. J. (2008). Orexin signaling mediates the antidepressant-like effect of calorie restriction. *J. Neurosci.* 28 3071–3075. 10.1523/jneurosci.5584-07.2008 18354010 PMC2713756

[B21] Marcantonio E. R. (2017). Delirium in Hospitalized Older Adults. *N. Engl. J. Med.* 377 1456–1466. 10.1056/NEJMcp1605501 29020579 PMC5706782

[B22] Maswood N. Young J. Tilmont E. Zhang Z. Gash D. M. Gerhardt G. A. (2004). Caloric restriction increases neurotrophic factor levels and attenuates neurochemical and behavioral deficits in a primate model of Parkinson’s disease. *Proc. Natl. Acad. Sci. U.S.A.* 101 18171–18176. 10.1073/pnas.0405831102 15604149 PMC539733

[B23] Melnyk M. Casey R. G. Black P. Koupparis A. J. (2011). Enhanced recovery after surgery (ERAS) protocols: time to change practice? *Can. Urol. Assoc. J.* 5 342–348. 10.5489/cuaj.11002 22031616 PMC3202008

[B24] Morgan J. I. Curran T. (1986). Role of ion flux in the control of c-fos expression. *Nature* 322 552–555. 10.1038/322552a0 2426600

[B25] Oresanya L. B. Lyons W. L. Finlayson E. (2014). Preoperative assessment of the older patient: a narrative review. *JAMA* 311 2110–2120. 10.1001/jama.2014.4573 24867014

[B26] Overton J. M. Williams T. D. (2004). Behavioral and physiologic responses to caloric restriction in mice. *Physiol. Behav.* 81 749–754. 10.1016/j.physbeh.2004.04.025 15234180

[B27] Patterson R. E. Laughlin G. A. LaCroix A. Z. Hartman S. J. Natarajan L. Senger C. M. (2015). Intermittent fasting and human metabolic health. *J. Acad. Nutr. Diet.* 115 1203–1212. 10.1016/j.jand.2015.02.018 25857868 PMC4516560

[B28] Paxinos G. Franklin K. (2001). *The Mouse Brain in Stereotaxic Coordinates*, 2nd Edn. San Diego: Academic Press.

[B29] Peng M. Zhang C. Dong Y. Zhang Y. Nakazawa H. Kaneki M. (2016). Battery of behavioral tests in mice to study postoperative delirium. *Sci. Rep.* 6:29874. 10.1038/srep29874 27435513 PMC4951688

[B30] Preston G. A. Lyon T. T. Yin Y. Lang J. E. Solomon G. Annab L. (1996). Induction of apoptosis by c-Fos protein. *Mol. Cell Biol.* 16 211–218. 10.1128/mcb.16.1.211 8524298 PMC230994

[B31] Prut L. Belzung C. (2003). The open field as a paradigm to measure the effects of drugs on anxiety-like behaviors: a review. *Eur. J. Pharmacol.* 463 3–33. 10.1016/s0014-2999(03)01272-x 12600700

[B32] Raats J. W. Steunenberg S. L. de Lange D. C. van der Laan L. (2016). Risk factors of post-operative delirium after elective vascular surgery in the elderly: a systematic review. *Int. J. Surg.* 35 1–6. 10.1016/j.ijsu.2016.09.001 27613124

[B33] Rayatnia F. Javadi-Paydar M. Allami N. Zakeri M. Rastegar H. Norouzi A. (2011). Nitric oxide involvement in consolidation, but not retrieval phase of cognitive performance enhanced by atorvastatin in mice. *Eur. J. Pharmacol.* 666 122–130. 10.1016/j.ejphar.2011.05.017 21620819

[B34] Riediger T. Bothe C. Becskei C. Lutz T. A. (2004). Peptide YY directly inhibits ghrelin-activated neurons of the arcuate nucleus and reverses fasting-induced c-Fos expression. *Neuroendocrinology* 79 317–326. 10.1159/000079842 15256809

[B35] Robinson T. N. Wu D. S. Pointer L. F. Dunn C. L. Moss M. (2012). Preoperative cognitive dysfunction is related to adverse postoperative outcomes in the elderly. *J. Am. Coll. Surg.* 215 12–17 discussion 17–18. 10.1016/j.jamcollsurg.2012.02.007 22626912 PMC3383613

[B36] Sestakova N. Puzserova A. Kluknavsky M. Bernatova I. (2013). Determination of motor activity and anxiety-related behaviour in rodents: methodological aspects and role of nitric oxide. *Interdiscip. Toxicol.* 6 126–135. 10.2478/intox-2013-20 24678249 PMC3967438

[B37] Silbert B. Evered L. Scott D. A. McMahon S. Choong P. Ames D. (2015). Preexisting cognitive impairment is associated with postoperative cognitive dysfunction after hip joint replacement surgery. *Anesthesiology* 122 1224–1234. 10.1097/aln.0000000000000671 25859906

[B38] Tucci V. Hardy A. Nolan P. M. (2006). A comparison of physiological and behavioural parameters in C57BL/6J mice undergoing food or water restriction regimes. *Behav. Brain Res.* 173 22–29. 10.1016/j.bbr.2006.05.031 16870275

[B39] Vasilevskis E. E. Han J. H. Hughes C. G. Ely E. W. (2012). Epidemiology and risk factors for delirium across hospital settings. *Best Pract. Res. Clin. Anaesthesiol.* 26 277–287. 10.1016/j.bpa.2012.07.003 23040281 PMC3580997

[B40] Velazquez F. N. Caputto B. L. Boussin F. D. (2015). c-Fos importance for brain development. *Aging* 7 1028–1029. 10.18632/aging.100862 26684501 PMC4712328

[B41] Vutskits L. Xie Z. (2016). Lasting impact of general anaesthesia on the brain: mechanisms and relevance. *Nat. Rev. Neurosci.* 17 705–717. 10.1038/nrn.2016.128 27752068

[B42] Wolf A. Bauer B. Abner E. L. Ashkenazy-Frolinger T. Hartz A. M. (2016). A comprehensive behavioral test battery to assess learning and memory in 129S6/Tg2576 mice. *PLoS One* 11:e0147733. 10.1371/journal.pone.0147733 26808326 PMC4726499

[B43] Zhu B. Dong Y. Xu Z. Gompf H. S. Ward S. A. P. Xue Z. (2012). Sleep disturbance induces neuroinflammation and impairment of learning and memory. *Neurobiol. Dis.* 48 348–355. 10.1016/j.nbd.2012.06.022 22776332 PMC3461115

