# Supplementary figures and images for: Acute Fasting Does Not Induce Cognitive Impairment in Mice
Source: Front Neurosci. 2019 Aug 26;13:896. doi: 10.3389/fnins.2019.00896 (PMC6718131; doi:10.3389/fnins.2019.00896)

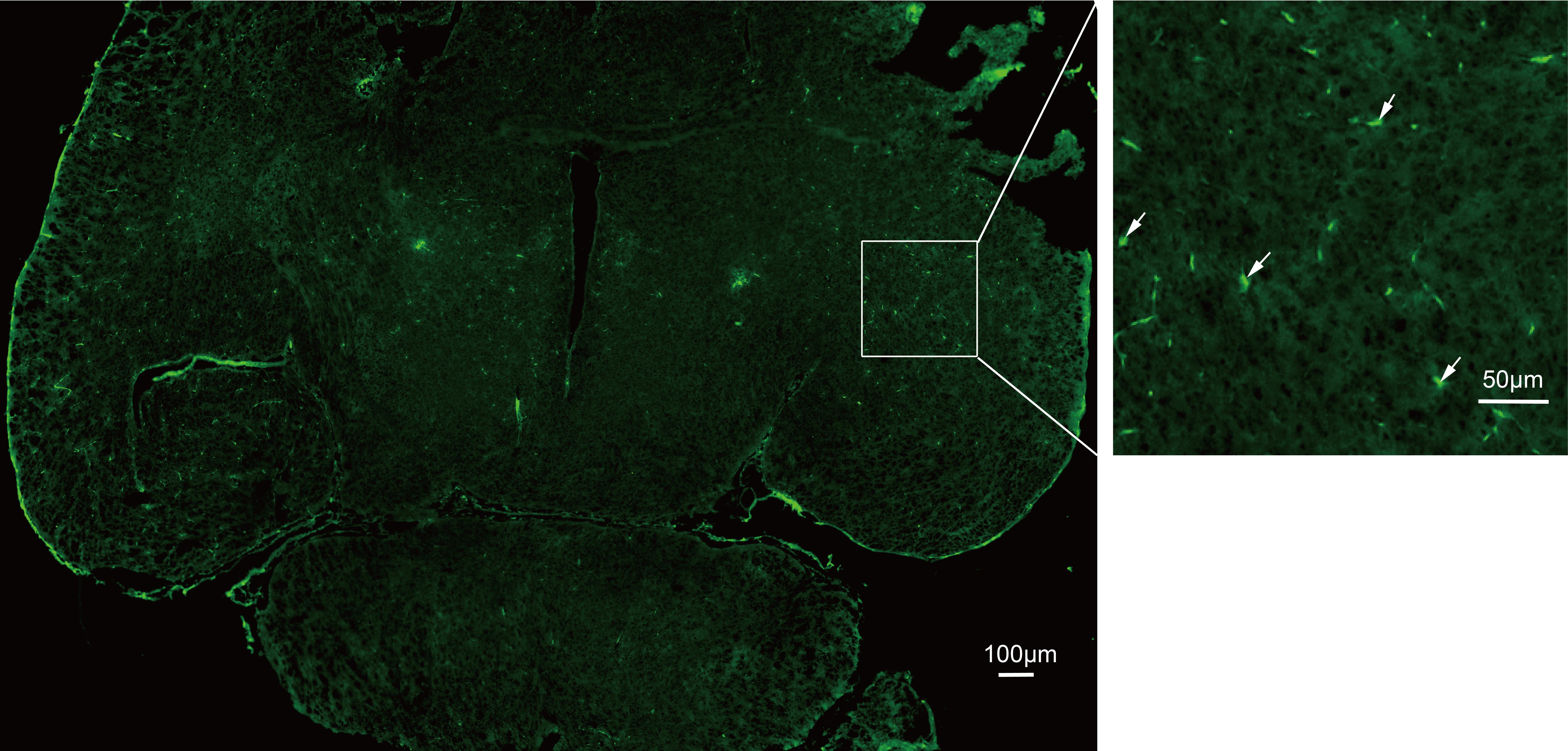

Supplement: FIGURE S1 — Positive control of immunohistochemistry stained with anti-cleaved caspase-3 antibody. The representative immunohistochemical staining is showing that sleep disturbance -induced cleaved caspase-3 expression distribute in the cortex of mice. The coronal section is located 1.2 ± 0.2 mm anterior from the bregma. The region of interest is outlined with a dash-dot line. Arrows indicate cleaved caspase-3-positive neurons. Scale bars, 100 μm (left image), 50 μm (right image). [file Image_1.JPEG]
